# The Content and Quality of Publicly Available Information About Congenital Diaphragmatic Hernia: Descriptive Study

**DOI:** 10.2196/30695

**Published:** 2021-10-19

**Authors:** Frank Coyle Soltys, Kimi Spilo, Mary C Politi

**Affiliations:** 1 Division of Newborn Medicine, Department of Pediatrics Washington University School of Medicine Washington University in St. Louis St. Louis, MO United States; 2 Division of Public Health Sciences Department of Surgery Washington University in St. Louis School of Medicine St. Louis, MO United States

**Keywords:** congenital diaphragmatic hernia, prenatal counseling, fetal care, online information, parental decision making

## Abstract

**Background:**

Congenital diaphragmatic hernia (CDH) diagnosis in an infant is distressing for parents. Parents often feel unable to absorb the complexities of CDH during prenatal consultations and use the internet to supplement their knowledge and decision making.

**Objective:**

We aimed to examine the content and quality of publicly available, internet-based CDH information.

**Methods:**

We conducted internet searches across 2 popular search engines (Google and Bing). Websites were included if they contained CDH information and were publicly available. We developed a coding instrument to evaluate websites. Two coders (FS and KS) were trained, achieved interrater reliability, and rated remaining websites independently. Descriptive statistics were performed.

**Results:**

Searches yielded 520 websites; 91 met inclusion criteria and were analyzed. Most websites provided basic CDH information including describing the defect (86/91, 95%), need for neonatal intensive care (77/91, 85%), and surgical correction (79/91, 87%). Few mentioned palliative care, decisions about pregnancy termination (13/91, 14%), or support resources (21/91, 23%).

**Conclusions:**

Findings highlight the variability of information about CDH on the internet. Clinicians should work to develop or identify reliable, comprehensive information about CDH to support parents.

## Introduction

With an incidence of 1:2500 live births, congenital diaphragmatic hernia (CDH) is a relatively common, yet complicated and potentially devastating diagnosis [1]. CDH can cause neurodevelopmental delays, chronic lung disease, gastroesophageal reflux, hearing loss, and even death [1]. As a result, parents whose fetus or newborn is diagnosed with CDH face decisions about extracorporeal membrane oxygenation (ECMO), management of long-term CDH complications, and potential plans for end-of-life care.

A diagnosis of CDH triggers numerous emotions, making it difficult for parents to absorb and process the information initially presented to them during a clinical visit [2-6]. Typically, parents receive this diagnosis and the complex information in a single prenatal visit. Many parents do not feel that one consultation provides enough time to learn about the diagnosis and its implications [6]. However, most parents want to engage in decisions regarding their baby’s care during this time, working with clinicians to support the decision-making process [7-10].

When met with uncertainty, parents often search for medical information outside of clinical encounters to make informed choices [11-13]. The internet is a popular resource for patients and families facing a difficult diagnosis such as CDH [11-14]. Despite its popularity, little is known about the comprehensiveness of internet-based CDH information [15].

This study aimed to evaluate the content and quality of internet-based information parents might find about CDH. Results could support the development or updating of websites to facilitate parental education and decision making about CDH.

## Methods

### Internet Searches

We conducted searches using 9 different terms on Google: “Congenital diaphragmatic hernia”, “CDH in a baby”, “Congenital diaphragmatic hernia surgery”, “Congenital diaphragmatic hernia NICU”, “Baby with stomach in chest”, “Baby with hole in diaphragm”, “CDH support for parents”, “Affording CDH/NICU costs”, and “CDH parent support website”. These search terms were chosen due to their use of lay-person terminology, and based upon clinical discussions with families of patients with CDH. We reviewed these terms with several practicing clinicians treating families facing this decision. We repeated searches on Bing until it was clear that the results produced the same websites. As most (91%) people do not look beyond the first page of search results [16], we included the first 3 pages for completeness.

Websites were included if they contained: (1) basic CDH information; (2) resources for patients with CDH or their parents; and (3) discussion boards, chat rooms, or social support information regarding CDH. Exclusion criteria included paid advertisements, legal sites, non-US sites, sites targeted to medical professionals, definition-only sites (ie, dictionary.com), sites requiring logins, and sites not about CDH.

We coded included sites’ content on the first page plus 2 clicks from the first page. Content linked to external sites was not coded. The study did not involve human patients, thus institutional review board approval was unnecessary.

### Website Coding

We developed a coding instrument of 133 items in the following categories: (1) basic definition or description of CDH; (2) prenatal care for CDH; (3) typical hospital course for patients with CDH; (4) ECMO procedure and complications; (5) CDH outcomes; (6) prenatal CDH surgery; (7) postnatal CDH surgery; and (8) financial, emotional, or personal support. This instrument was reviewed for accuracy and completeness by 2 neonatologists with experience in treating CDH.

The first 7 websites were coded by 2 raters (FS and KS) to check for consistency in coding. Cohen κ was 0.75 with a 79% agreement at this stage. The 2 raters met, discussed discrepancies, and reached consensus, revising the codebook where necessary. Once Cohen kappa showed a high level of agreement (κ>0.80; agreement >90%), remaining sites were divided and scored by 1 of the 2 coders (FS or KS). The 2 coders remained in contact throughout the process to ensure consistency. We analyzed the data using descriptive statistics.

## Results

### Internet Searches

The searches yielded 520 websites. A total of 368 websites were excluded initially because they were duplicates (n=264, 71.7%), advertisements (n=91, 24.7%), or scholarly articles intended for medical professionals (n=37, 10.1%). Of the remaining 152 websites, 61 (40.1%) did not meet additional inclusion criteria about CDH content. Of the 91 analyzed sites, most were developed by academic medical centers (n=53, 58%), general medical knowledge sources (n=10, 11%), or nonprofit organizations (n=10, 11%).

### Website Coding

Most websites described basic CDH information (86/91, 95%), types of CDH (52/91, 57%), implications for prenatal care (55/91, 60%), or variation in clinical acuity (56/91, 62%; Table 1). Websites infrequently mentioned various complications of CDH. Many did not mention treatment options such as pregnancy termination, palliative care, or a compassionate delivery. Only 13/91 (14%) sites mentioned pregnancy termination as an option. Only 4/91 (4%) discussed the possibility of palliative care or compassionate delivery. There was a paucity of discussion around financial, emotional, or informational support for the family.

**Table 1:** Content of CDH^a^ websites (N=91).

			Number of websites (%)
**CDH information**			
	Gave description or definition of CDH	86 (95)	
	Mentioned types of CDH	52 (57)	
	Discussed how CDH is diagnosed	74 (81)	
	Discussed prenatal care for CDH	55 (60)	
	Mentioned admission to the neonatal intensive care unit	77 (85)	
	Discussed potential need for a breathing tube/intubation	73 (80)	
	Discussed postnatal surgery	79 (87)	
	Discussed variation in clinical acuity	56 (62)	
	Discussed possibility of death from CDH	60 (66)	
**Potential complication of CDH**			
	Discussed risk of neurodevelopmental delays from CDH	45 (49)	
	Discussed risk of chronic lung disease	46 (51)	
	Discussed risk of hearing difficulties due to CDH	26 (29)	
	Discussed risk of gastroesophageal reflux	49 (54)	
	Discussed the potential for the hernia to recur	19 (21)	
	Discussed risk of failure to thrive/inability to gain weight	35 (38)	
**Treatment option**			
	Discussed potential for ECMO^b^	66 (73)	
	If mentioned ECMO, site described complications of ECMO	21 (23)	
	Discussed the possibility of prenatal surgery	35 (38)	
	Discussed possibility of termination of pregnancy	13 (14)	
**Support system information**			
	Contained additional reading material for parents regarding the diagnosis of CDH	24 (26)	
	Discussed financial support	15 (16)	
	Discussed housing options while in the neonatal intensive care unit	12 (13)	
	Contained emotional/personal support resources for families	21 (23)	
	Provided information regarding mental health resources	9 (10)	

^a^CDH: congenital diaphragmatic hernia.

^b^ECMO: extracorporeal membrane oxygenation.

## Discussion

Access to comprehensive, accurate information about CDH is critical to supplement clinical visits and support parents with infants with a CDH diagnosis. We examined the quality of available CDH information on the internet. Many websites described basic information about CDH, including a description of CDH and possible medical interventions. However, few websites described possible negative outcomes, complications, and care options aside from full medical interventions.

When searching for CDH information, families can become overwhelmed with the number of results obtained. Our study used search terms and phrases similar to what a typical family might use. We found numerous websites that were not accessible or relevant to families, highlighting the difficulty in conducting generalized searches about CDH. Families could become frustrated when attempting to find comprehensive and reliable information; clinicians could supply a list of high-quality websites for parents. The use of websites with quality information about CDH hosted by reputable institutions or organizations can support families.

The scarcity of discussion around palliative care, compassionate delivery, and pregnancy termination should be noted, as these are reasonable options for families. One CDH parent advocacy website mentioned the lack of in-person discussion about palliative care or compassionate delivery [17]. At a time when parents desire involvement in care, they should have access to information about all reasonable options for their infants. Parents should also be aware of the complications of CDH to be as informed as possible when making care decisions.

These data should be considered within the context of some limitations. Searches were completed once (October 2019) with a single update (January 2020). Websites could have edited information after the search and coding process. We used 2 popular search engines (Google and Bing), but families could find additional sites not identified. We also used experience with previous CDH families to guide search term creation; however, parental input on search terms may have yielded different results. We excluded social media sites that required a login, although some social media sites could provide information or support through peer groups. We included information 2 clicks from the main page, but parents could go further into the sites. We did not analyze whether the information presented on websites was clear; future studies can use tools such as the Clear Communication Index or the Patient Education Materials Assessment Tool (PEMAT) to analyze specific sites once sources are identified and considered for use with patients. Finally, the coders were able to traverse the websites with relative ease, but parents might not be as savvy with the internet, and thus results could overestimate information available.

This study highlights a need for more comprehensive websites with information about CDH. Institutional or clinic-based materials might better support families than internet resources as families navigate through CDH information seeking and decision making.

## Abbreviations

CDH: congenital diaphragmatic hernia
ECMO: extracorporeal membrane oxygenation
PEMAT: Patient Education Materials Assessment Tool
